# Journal- or article-based citation measure? A study of academic promotion at a Swiss university

**DOI:** 10.12688/f1000research.26579.1

**Published:** 2020-10-01

**Authors:** Nicole Steck, Lukas Stalder, Matthias Egger

**Affiliations:** 1 Institute of Social and Preventive Medicine, University of Bern, Bern, Switzerland; 2 Dean's office, Medical Faculty, University of Bern, Bern, Switzerland; 3 Department of Population Health Sciences, Bristol Medical School, University of Bristol, Bristol, UK

**Keywords:** Relative Citation Ratio, Journal Impact Factor, DORA Declaration

## Abstract

In academia, decisions on promotions are influenced by the citation impact of the works published by the candidates. The Medical Faculty of the University of Bern used a measure based on the journal impact factor (JIF) for this purpose: the JIF of the papers submitted for promotion should rank in the upper third of journals in the relevant discipline (JIF rank >0.66). The San Francisco Declaration on Research Assessment (DORA) aims to eliminate the use of journal-based metrics in academic promotion. We examined whether the JIF rank could be replaced with the relative citation ratio (RCR), an article-level measure of citation impact developed by the National Institutes of Health (NIH). An RCR percentile >0.66 corresponds to the upper third of citation impact of articles from NIH-sponsored research. We examined 1525 publications submitted by 64 candidates for academic promotion at University of Bern. There was only a moderate correlation between the JIF rank and RCR percentile (Pearson correlation coefficient 0.34, 95% CI 0.29-0.38). Among the 1,199 articles (78.6%) published in journals ranking >0.66 for the JIF, less than half (509, 42.5%) were in the upper third of the RCR percentile. Conversely, among the 326 articles published in journals ranking <0.66 regarding the JIF, 72 (22.1%) ranked in the upper third of the RCR percentile. Our study demonstrates that the rank of the JIF is a bad proxy measure for the actual citation impact of individual articles. The Medical Faculty of University of Bern has signed DORA and replaced the JIF rank with the RCR percentile to assess the citation impact of papers submitted for academic promotion.

## Introduction

In academia, decisions on promotion to senior positions are influenced by the work published by the candidate. The assessment of publication lists should be systematic, using standardized criteria, and straightforward ^1^. Bibliometric measures such as the journal impact factor (JIF) or its rank within a given field meet this requirement. The JIF or its rank reflect citations to all articles published in the journal, rather than citations to the article submitted by the candidate. Of note, it was initially created as a tool to help librarians identify the journals they should subscribe to, and not as a measure of the scientific quality or impact of research ^2^. In 2013, the San Francisco Declaration on Research Assessment (DORA) was published with the aim to improve the way research output is evaluated ^2^. Research should be assessed on its own merits rather than based on the journal in which it is published. The first general recommendation of DORA says “Do not use journal-based metrics, such as Journal Impact Factors, as a surrogate measure of the quality of individual research articles, to assess an individual scientist’s contributions, or in hiring, promotion, or funding decisions.” ^2^. As of May 2020, more than 1,900 organizations and over 15,000 individuals have signed the DORA declaration.

There is thus growing consensus that the JIF is not a good measure to assess individual research papers. Efforts have been underway for several years to find a practical measure by which the citation impact of papers can be individually evaluated ^3– 7^. In 2016 Hutchins *et al.* ^8^, presented the Relative Citation Ratio (RCR), an article-level measure of citation impact which compares the citations to the article of interest with the articles in the network of co-cited articles ^8^. The RCR is benchmarked to National Institutes of Health (NIH)-funded research ^9^: an article with an RCR equal to 1.0 is at the median for NIH-funded articles in this year ^10^. The NIH provides access to the RCR and percentile of papers indexed in the PubMed bibliometric database on a website ^11^. Several studies used the RCR to assess the citation impact of researchers, for example, vascular surgeons within the NIH ^12^, biomedical scientists in one country ^13^ or papers from scientific publications produced by the United States Food and Drug Administration (FDA) ^14^.

The University of Bern signed the DORA declaration in January 2016. Therefore, the Medical Faculty decided to review its practice for internal promotion, where the assessment of citation impact was based on the rank of the JIF ^15^. In 2018, a working group of the Medical Faculty examined whether the RCR could replace the ranking of the JIF as a decision-making aid for hiring, tenure and promotion decisions. The present study aimed to investigate the effects of switching from journal-based JIF-ranking to the RCR in the assessment of the papers submitted by candidates.

## Methods

### Academic promotion

In medical faculties in Switzerland, the habilitation degree and promotion to associate professor are essential steps in an academic career. The habilitation degree was introduced in the first half of the 19th century to ensure the quality of academic teaching and research at German universities. Today the habilitation is a post-doctoral qualification, which is required for independent teaching and supervision of doctoral students and to obtain an associate or full professorship in many European countries, including Switzerland ^16^. At the University of Bern, the degree is conferred based on an application which includes a list of papers and a summary of the work highlighting the applicant’s contributions in research and teaching. An academic committee reviews the application, and the candidate presents and discusses his/her research at a faculty meeting. A similar process is followed for promotion to associate professor.

Until 2019, the faculty used the rank of the JIF ^15^ for its assessment of applications for promotion to habilitation or associate professorships, and also The Journal Citation Reports (JCR) ^15^ rank journals based on the JIF within subject categories, for example, oncology, surgery or nursing. Per university regulations ^17^, candidates for habilitations needed at least ten original articles with a JIF rank in the upper third of the relevant discipline, and among the ten papers four as first or last author. The successful habilitation is a prerequisite for promotion to associate professor. The guidelines for promotion to associate professor required at least six additional original papers published in journals of the upper third of the JIF-based ranking, with at least three as first or last author ^18^.

### Study sample and data sources

The Dean’s office of the Medical Faculty of the University of Bern compiled the publication lists submitted by a randomly selected 34 candidates for habilitation and 30 candidates for associate professor in 2017 and 2018. For each paper, we recorded the JIF of that year and its ranking in the corresponding field. The data were obtained from the JCR of Clarivate Analytics ^15^. The Relative Citation Ratio (RCR) and the RCR percentile were obtained from the iCite tool ^11^. Since the results for the papers submitted by candidates for the habilitation degree and for associate professorship were similar, we combined the data in the analysis.

### Analysis

We assessed the papers submitted by the candidates and the number of first- or last-author papers. We calculated the number with a JIF ranking in the upper third (the cutoff defined in the regulations) and the number with an RCR percentile >66%. To visualize the distribution of RCR percentile and JIF ranking by candidate, we used beam plots ^19^ and kernel density estimation (Epanechnikov distribution, bandwith=5.0). We examined the relation between RCR percentiles and JIF rank in scatterplots and calculated the Pearson correlation coefficient and its confidence interval.

The calculation of the RCR and its percentile requires the article of interest to be cited so that these citations can be compared with those received by the articles in the co-citation network ^8^. Papers in the second year after publication or more recent papers with five or more citations receive a provisional RCR ^11^. We included both articles with definitive and provisional RCRs in the main analysis. For each candidate with articles from both categories, we calculated the difference between the articles with definitive RCR and all articles, including provisional RCRs, and combined the differences using random-effects meta-analysis. All statistical analyses were done in Stata version 15 (StataCorp, College Station, TX, USA).

## Results

The 64 candidates submitted 1,903 original articles, including 801 (42.1%) first- or last-author papers. A total of 134 papers (7.0%) had no JIF, and 328 (17.2%) had no RCR; 378 papers had to be excluded. A total of 1,525 articles were included in the analyses, including 625 (41.0%) first- or last-author papers and 223 (14.6%) articles with a provisional RCR. At the time of the download of the bibliometric data (12 September 2018) the total number of citations to the 1,525 articles was 45,119.

### The relation between journal rank and article RCR

**Figure 1.**
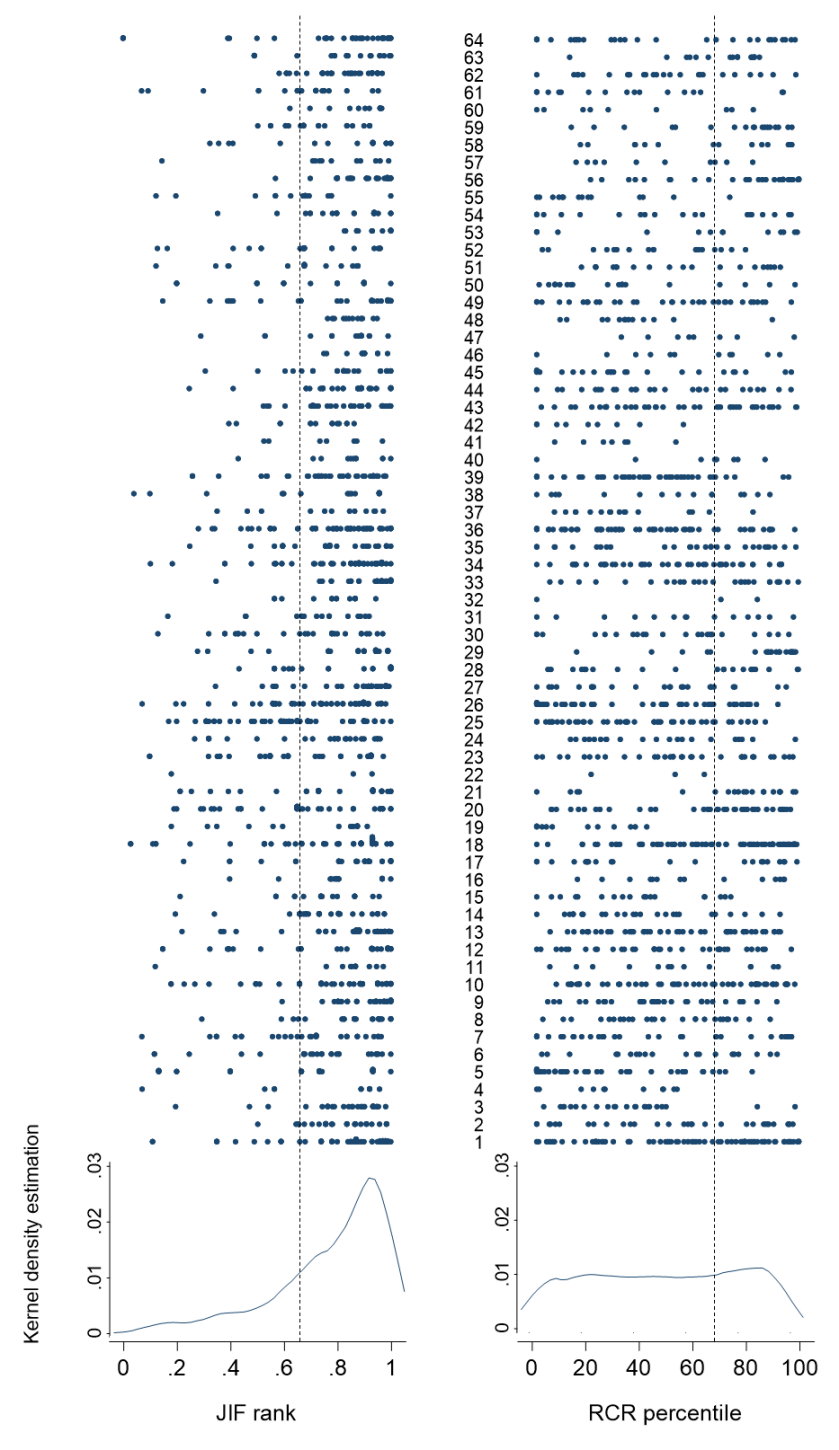
Beam plots and Kernel density estimation of JIF rank and RCR percentile of all original publications submitted by candidates for academic promotion at the University of Bern. JIF rank (left panel) and RCR percentile (right panel) are shown for each article submitted by candidates for habilitation (1-34) and associate professorship (35-64). Each candidate corresponds to one row. Kernel density estimation (epanechnikov, bandwith=5.0) for JIF rank (left panel) and RCR percentile (right panel) are shown below. The broken lines show rank 0.66 (left panel) and RCR percentile 66 (right panel).

The beam plot in Figure 1 shows, row-wise for each candidate, the JIF ranking and RCR percentile of all 64 candidates. The kernel density estimation at the bottom of Figure 1 shows the differences in distribution of JIF ranking and RCR percentiles. As expected from the university regulations, the majority of papers were in the upper third of the JIF rank for all candidates. In contrast, they were more evenly distributed across percentiles of the RCR. Of note, some candidates had few or no articles above the 66 ^th^ percentile of the RCR. Overall, 1,199 (78.6%) of 1,525 papers had a JIF rank in the upper third, and 581 (38.1%) had an RCR percentile above 66. Among the 625 first- and last-author papers, 489 (78.2%) had a JIF rank in the upper third, and 233 (37.3%) had an RCR percentile above 66. The beam plot and the kernel density estimation for first- and last-author papers was similar to the plot for all papers (see *Extended data:* Figure S1 ^20^).

**Figure 2.**
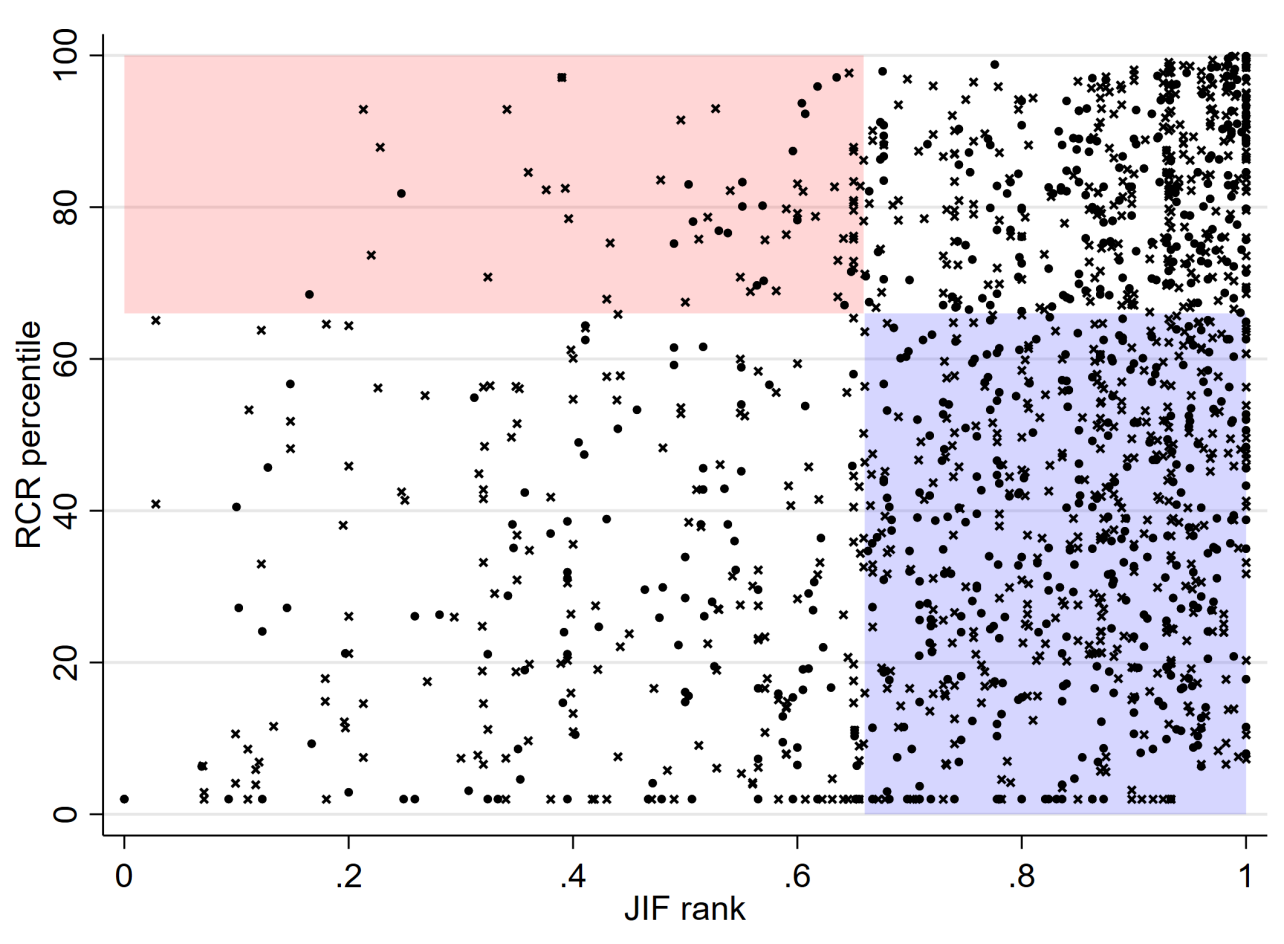
Scatter plot of RCR percentile against JIF rank of publications submitted by candidates for promotion at the University of Bern. Publications are shown as point or cross for candidates for habilitation and associate professorship, respectively. Cutoffs of 0.66 for the JIF rank (as per university regulations) and 66 for the RCR percentile define four quadrants. The pink top-left quadrant shows the publications that have an RCR percentile >66 but were published in a journal with a JIF ranking <0.66. The blue quadrant shows the papers published in a journal with a JIF rank >0.66 but had an RCR percentile <66.

Figure 2 shows a scatterplot of the RCR percentile against the JIF ranks of all 1,525 papers submitted by applicants for habilitation and candidates for the associate professorship with data on both indicators. The correlation coefficient was 0.34 (95% CI 0.29 to 0.38). The scatter plot is divided into for quadrants by cutoffs 0.66 (for JIF rank) and 66 (for RCR percentile). Among the articles published in journals with a JIF ranking in the upper third, 57.5% (690 of 1,199) did not have an RCR percentile above 66 (blue quadrant in Figure 2). Conversely, 22.8% (72 of 326) of articles published in journals with a JIF rank in the lower two thirds (<0.66) had an RCR percentile above 66 (pink quadrant in Figure 2). The results for first- and last-author papers were similar: the correlation coefficient was 0.31 (95% CI 0.24 to 0.38), and the percentages of papers in the blue and pink quadrants were 59.1% (289 of 489) and 24.3% (33 of 136), respectively ( *Extended data: Figure S2* ^20^).

### Definitive versus provisional RCRs

**Figure 3.**
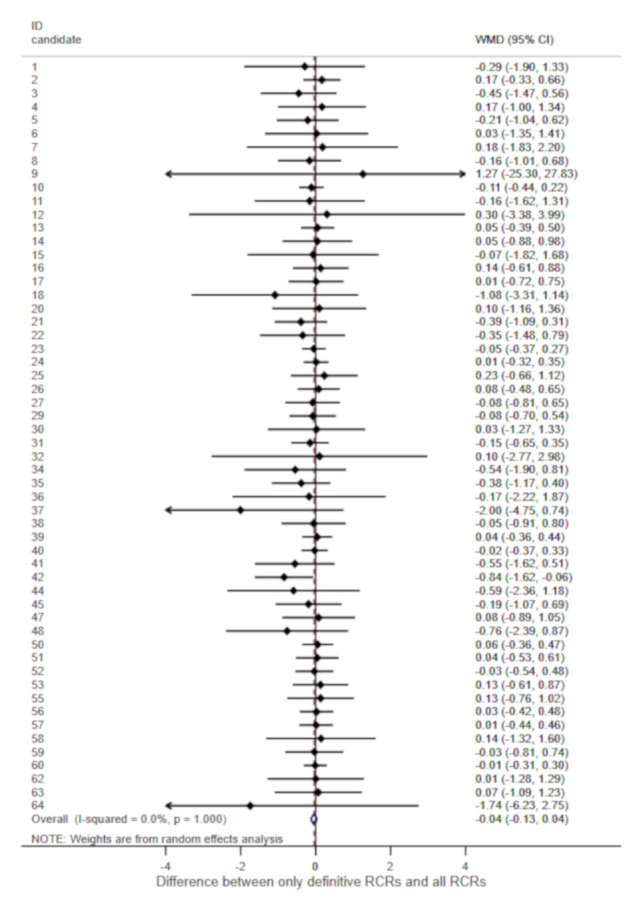
Meta-analysis of differences between means of definitive RCRs and all RCRs (definitive and provisional). For each candidate submitting articles with definitive and provisional RCRs the weighted mean difference (WMD, filled diamonds) and its 95% confidence interval (horizontal line) were calculated. The estimates were combined in a random-effects meta-analysis model. The empty diamond at the bottom shows the combined estimates from the meta-analysis of all candidates.

In total 57 candidates had both definitive and provisional RCRs ( Figure 3). The meta-analysis of the differences between definitive and all RCRs across candidates gave an overall weighted mean difference of -0.04 (95% CI -0.13 to 0.04). There was thus no evidence of a systematic bias due to provisional RCRs, and no heterogeneity between candidates (I squared 0.0%).

## Discussion

This analysis of papers submitted to promotion committees at a Swiss university illustrates that the rank of the journal’s impact factor within its discipline is a bad proxy measure of the citation impact of individual articles. Many articles published in higher impact journals were cited less than their companion papers in the co-citation network. Whereas the majority of the papers submitted by candidates for the habilitation or an associate professorship were, by university regulation, published in journals with a JIF that ranked in the upper third of its field, only about 40% of these papers had an RCR percentile in the upper third. Furthermore, 20–25% of the papers that did not meet the requirement for the JIF rank (below 0.66) were, in fact, more impactful than their peers in the co-citation network. Our study thus confirms the findings of the RCR developer’s analysis of 80,000 papers: “Though journals with the highest impact factors have the highest median RCR, influential papers can be found in virtually all journals” ^8^. Unsurprisingly, the correlation between the journal-based measure, the JIF, and the article-based measure, the RCR, was weak.

Several previous studies used the RCR to assess the citation impact of different groups of researchers ^12– 14^. To the best of our knowledge, this is the first study investigating differences between a journal-based metric and an article-based metric of citation impact in the context of academic promotion. We used a large “real-world” dataset of over 1,500 papers submitted by candidates for academic promotion at a large Swiss medical faculty. Our results provide further empirical evidence supporting the San Francisco Declaration on Research Assessment (DORA) ^2^, and challenges the practices at a Swiss university that relied inappropriately on the JIF. The results indicate that moving from a journal-based measure to an article-based measure is feasible. Indeed, the regulations of the Medical Faculty at the University of Bern have since been revised. The new regulations state that the assessment of candidates must follow the DORA principles. The committee analysing the papers should examine the novelty of the research question, the suitability of the methods, the interpretation of results and their relevance to the field. The evaluation should be based on the scientific content of the work. Article-based impact measures or qualitative indicators for the impact of the research (e.g. influence on policy and practice) may complement the assessment. Specifically, the regulations state that two or more of the papers with first or last authorship should have an RCR of 1 or higher. Also, the regulations explicitly state that “the journal and its impact factor will not be considered” ^21^. The regulations for associate professor and titular professor were revised in the same spirit. They also refer to DORA ^2^.

The RCR is based on citations and shares all the limitations of using citations as a proxy for impact. For example, the number of citations is influenced by factors unrelated to the quality of the research. The impact outside academia, including for political decision-making, is not well captured by citations ^22– 24^. Furthermore, unlike the JIF, the RCR requires time to allow citations to the article of interest to appear in the literature, which may limit its use in the context of academic promotion. In our study, only 328 of 1,903 (17.2%) had to be excluded because no RCR was available. Furthermore, within candidates, the inclusion of provisional RCRs of recent papers did not influence their mean RCR, indicating that provisional RCRs can be included in assessments. Of note, the developers of the RCR have shown that the RCR is usually very stable after one year ^11^. The RCR is based on Medline and therefore not suitable for assessing non-biomedical literature ^10^, for example, on medical ethics or teaching.

Moreover, when using the RCR, it should not be forgotten that the reference is the papers financed by the NIH ^8^. While an RCR of 1.0 corresponds to the median of the citations of NIH-funded articles, the median of all papers has an RCR of around 0.37 ^11^, subject to annual fluctuations. Another important point of criticism regarding the RCR is that “papers may be penalized rather than rewarded for receiving interdisciplinary citations” ^25^. If a paper from a low-citation field is published in a journal from a high-citation field, this could reduce its RCR ^25^. However, a comparison with other bibliometric indicators did not support this criticism ^26^. Also, the developers of the RCR found a good agreement between metric and expert reviewer scores ^8^.

In conclusion, we hope that our study will serve as a model to other researchers who, in the spirit of the DORA ^2^ intend to challenge research assessment practices at medical and other faculties that rely inappropriately on Journal Impact Factors and contribute to promoting best practice that focuses on the value and influence of specific research outputs.

## Data availability

### Underlying data

The original raw data is composed of personal data of individuals applying for academic promotion and can therefore not be shared. The data without the identifying variables is available:

Open Science Framework: Journal- or article-based citation measure? A study of academic promotion at a Swiss university, https://doi.org/10.17605/OSF.IO/H7SKN ^20^.

### Extended data

Open Science Framework: Journal- or article-based citation measure? A study of academic promotion at a Swiss university, https://doi.org/10.17605/OSF.IO/H7SKN ^20^.

This project includes the following extended data:

- **Figure S1. Beam plots and Kernel density estimation of JIF rank and RCR percentile of first- and last-author publications submitted by candidates for academic promotion at the University of Bern.** JIF (left panel) and RCR percentile (right panel) are shown for each article submitted by candidates for habilitation (1-34) and associate professorship (35-64) as first or last author. Each candidate corresponds to one row. Kernel density estimation (epanechnikov, bandwith=5.0) for JIF rank (left panel) and RCR percentile (right panel) are shown below. The broken lines show rank 0.66 (left panel) and RCR percentile 66 (right panel).
- **Figure S2. Scatter plot of RCR percentile against JIF rank of publications submitted by candidates for promotion at the University of Bern as first or last authors.** Publications are shown as point or cross for candidates for habilitation and associate professorship, respectively. Cutoffs of 0.66 for the JIF rank (as per university regulations) and 66 for the RCR percentile define four quadrants. The pink top-left quadrant shows the publications that have an RCR percentile >66 but were published in a journal with a JIF ranking <0.66. The blue quadrant shows the papers published in a journal with a JIF rank >0.66 but had an RCR percentile <66.

Data are available under the terms of the Creative Commons Zero “No rights reserved” data waiver (CC0 1.0 Public domain dedication).
