# Supplementary material for: Journal- or article-based citation measure? A study of academic promotion at a Swiss university
Source: F1000Res. 2020 Oct 1;9:1188. [Version 1] doi: 10.12688/f1000research.26579.1 (PMC7573719; doi:10.12688/f1000research.26579.1)
Supplement: Peer review history

# Peer review: Reviewer response for version 1

**DOI:** 10.5256/f1000research.29342.r72324

**Published:** 2020-10-29

**Authors:** Leonhard Held, Eva Furrer

**Affiliations:** 1 Epidemiology, Biostatistics and Prevention Institute (EBPI), Center for Reproducible Science (CRS), University of Zurich, Zurich, Switzerland

**General comments**

 This is an interesting paper that compares journal- with article-based citation measures for the promotion of Swiss academics. The results from this analysis seem to have been used as a basis to change the current system for academic proportion at the University of Berne.

 In the following we outline some aspects of the present study that could be improved to further justify its use for decision making. Some additional discussion of other promotional criteria related to teaching, academic service (such as peer review) and more general contributions for the benefit of society (e.g. software, policy briefs, open data etc) could also be added.

 A consistent use of either “paper” or “article” would add more clarity to the paper.

**Specific comments**

**Introduction, 2nd paragraph:**

- *“The RCR is benchmarked to National Institutes of Health (NIH)-funded research: an article* *with an RCR equal to 1.0 is at the median for NIH-funded articles in this year.” *
  Change “this year” to “the same year”

**Methods, Academic promotion, 2nd paragraph**

- *“Until 2019, the faculty used the rank of the JIF for its assessment of applications for promotion* *to habilitation or associate professorships, [...]” *
  Surely the applications have been assessed in other ways too and the JIF has only been used to quantify the publication output of the candidates?
- *“Per university regulations, candidates for habilitations needed at least ten original articles”*
  Change “habilitations” to “habilitation”
- *“The successful habilitation is a prerequisite for promotion to associate professor.”*
  This has been mentioned in the paragraph above and can be deleted.

**Study sample and data sources**

- *“The Dean’s office of the Medical Faculty of the University of Bern compiled the publication* *lists submitted by a randomly selected 34 candidates for habilitation and 30 candidates for* *associate professor in 2017 and 2018.”*
  Change “submitted by a randomly selected” to “submitted by randomly selected”.
  This paragraph suggests that the study sample was not just a simple convenience sample. More details are needed on how this random sample has been obtained: how large was the underlying source population, why the sample size of 64 and how exactly was the sampling done?
- *“For each paper, we recorded the JIF of that year and its ranking in the corresponding field.”*
  Change “that year” to “the corresponding year”
- *“Since the results for the papers submitted by candidates for the habilitation degree and for*
*associate professorship were similar, we combined the data in the analysis.”*
  What does similar mean here exactly?

**Analysis**

 The following issues need attention:

- Does a study protocol (with pre data collection analysis plan) exist?
- The data provided on OSF is not easy to read in - please separate explanation of variables from the actual data file, preferably not in Excel.
- For “Promotion=AssocProf” the variables (“First_author”, “Last_author”) sometimes have the values (1, .) resp. (., 1) where “.” presumably means “missing”. What is the difference to (1, 0) resp. (0, 1) for “Promotion=Habil”? If a candidate is a first author then she/he cannot be a last author and vice versa.
- The code of the analysis should be made available to make the analysis reproducible.
- Would it be possible and useful to follow the STROBE reporting guidelines on observational studies to some extent?
- Figure 1: The use of kernel smoothing along with the beam plots has the disadvantage that it extends beyond the domain boundaries (0-1). Also the y-scale of the left plot (kernel density estimation of JIF rank) is wrong, as the density does not integrate to unity. A simple histogram would provide the same information and is methodologically more appropriate.
- One of the key results of the paper is the weak positive correlation between JIF rank and RIC percentile. However, in view of the scatter plot in Figure 2 a Spearman or Kendall rank correlation seems more appropriate than a Pearson correlation.
- For the meta analysis it is written “For each candidate with articles from both categories, we calculated the difference between the articles with definitive RCR and all articles, including provisional RCRs, and combined the differences using randomeffects meta analysis.” This seems to indicate that for each candidate an average RCR of all articles with definitive RCR has been subtracted from an average RCR of all definitive and provisional articles. In Figure 3 it is mentioned that a weighted mean difference has been used. This needs to be clarified in more detail. Moreover, it needs to be made clearer to what purpose the meta analysis is used. All methods for which results are provided in the Results section should be described in the methods section, specifically the I-squared and the corresponding test. Figure 3 would profit from an indication which side corresponds to higher RCR for articles including only definitive RCR and which side for higher RCR for articles including definitive and provisional RCR.
- We are also wondering if the Hartung-Knapp or the traditional DerSimonian-Laird method is used for random effects meta-analysis, Hartung-Knapp is known to perform better, see http://www.biomedcentral.com/1471-2288/14/25.
- In the Results Section we learn that missing values are present. A discussion in the Methods Section on the treatment of missing values is needed. Specifically, an explanation why no imputation methods have been considered and some discussion of possible bias this may have caused.

**Results, 1st paragraph**

- 378 papers had to be excluded: how many authors does this affect and on average how many papers per author?

**The relation between journal rank and article RCR**

- A more consistent section header would be “The relation between JIF rank and article RCR percentile”.

**1st paragraph**

- *“The beam plot in Figure 1 shows, row-wise for each candidate, the JIF ranking and RCR* *percentile of all 64 candidates.”*
  The plot shows the JIF ranking and RCR of all papers of a candidate not of the candidates themselves.

***2nd paragraph***

- 72 divided by 326 is 22.1% not 22.8%.

**Definitive versus provisional RCRs**

- *“In total 57 candidates had both definitive and provisional RCRs (Figure 3).”*
  Again, it is not the candidates but their papers that have RCRs.

***Discussion, 2nd paragraph***

- *“Our results provide further empirical evidence supporting the San Francisco Declaration on* *Research Assessment (DORA), and challenges the practices at a Swiss university that* *relied inappropriately on the JIF.”*
  Change “challenges” to “challenge”, “practices” to “former practices”
  We think this paragraph could be made clearer how exactly the results of this study influenced the faculty board in their decision to change practices. Was the study done before the decision and was the decision conditional on its results?

**3rd paragraph**

- *“For example, the number of citations is influenced by factors unrelated to the quality of the* *research.”*
  Can you give a reference for this statement?
- *“The RCR is based on Medline and therefore not suitable for assessing non-biomedical literature* *, for example, on medical ethics or teaching.”*
  It would be better to stick with the term “Pubmed” and not use Medline, this will be confusing to readers not familiar with biomedical research.

**4th paragraph**

- *“Moreover, when using the RCR, it should not be forgotten that the reference is the papers* *financed by the NIH.”*
  Change “the reference is the papers” to “the reference are the papers”.

**Last paragraph**

- You imply that the change in Berne was due to the initiative of researchers. Is this the case or was it a management decision? Hence the public you want to address with this article are not individual researchers but researchers who are now in managerial positions, right?

Is the work clearly and accurately presented and does it cite the current literature?

Yes

If applicable, is the statistical analysis and its interpretation appropriate?

Partly

Are all the source data underlying the results available to ensure full reproducibility?

Yes

Is the study design appropriate and is the work technically sound?

Yes

Are the conclusions drawn adequately supported by the results?

Yes

Are sufficient details of methods and analysis provided to allow replication by others?

Partly

Reviewer Expertise:

LH: Biostatistics, Reproducibility and Replicability, Bayesian Biostatistics, Infectious Disease Epidemiology EF: Biostatistics, Reproducibility, Scientific Rigor and Science Policy

We confirm that we have read this submission and believe that we have an appropriate level of expertise to confirm that it is of an acceptable scientific standard, however we have significant reservations, as outlined above.

# Peer review: Reviewer response for version 1

**DOI:** 10.5256/f1000research.29342.r72318

**Published:** 2020-10-19

**Authors:** Bernd Pulverer

**Affiliations:** 1 European Molecular Biology Organization (EMBO), Heidelberg, Germany

This is a very valuable analysis underlying an important research assessment policy change at the University of Bern: a switch from assessing the top third research papers published by candidates for academic promotion as classified by the Clarivate 'Journal Impact factor' to the 'Relative Citation Ratio' (RCR), which uses a subject/citation network weighed algorithm. The analysis is based on 1,525 paper by 64 individuals and represents a sufficiently large dataset to yield meaningful results. The article concludes that RCR and JIF based rankings of papers do not show a significant correlation. This is important, but the authors should be careful not to conclude from the presented data that RCR is necessarily a good metric for academic performance.

 I suggest the authors consider enhancing the study by addressing the following points:

**Major:**

1. The papers analyzed are from 2017/18 and the analysis from 2018. I suggest to re-run the citation counts for 2020. This will ensure that citation rates to most medical papers have peaked and will allow the authors to extend the comparison between JIF and RCR to a third important metric: actual citations to the papers analyzed (I suggest using a fixed time window of e.g. 18 months).
2. Given the dramatic differences in the assessment by RCR vs. JIF and the policy change by Bern, it would be helpful to others considering adopting this policy change to specify how many candidates would have been reclassified in the academic assessment as a result of this change.
3. The RCR is presented without any critical discussion as the clear article-level indicator of choice. Please discuss limitations at least briefly e.g. the citation network is heavily based on reference lists, which can in principle be 'gamed' by authors to increase RCR rankings.
4. On p. 5 the authors conclude that candidates were more evenly distributed across percentiles using the RCR. This may not affect habilitation and academic performance evaluation, but it may affect hiring, where rank lists are used, since candidate ranking appear less pronounced. 

**Minor:**

1. The Abstract refers to a 'moderate correlation', while p.7 refers to a 'weak' correlation. fig shows a very weak correlation at best.
2. It would be helpful to specify in more detail what disciplines were covered and if any differences were noted between subject areas in the analyzed dataset.
3. JIF and JIF rank within a Clarivate attributed field are treated as similarly problematic: I suggest to note that field ranking is even more problematic as the journals included are v. patchy e.g. general interest journals are not included.
4. It would be useful to briefly describe how the faculty plans to implement this significant policy change, especially during the transition period. Equally, to describe in more detail if other attributes are considered such as teaching quality (since 'habilitation' is described as a degree to 'ensure the quality of academic teaching and research' (p. 3).
5. V. minor: add a comma for 1,525, in the abstract; add 'in principle' to line 6, p. 3; add 'the decl. states that' to line 14, p.3; change 'had no JIF' to 'were publ. in jnl. without JIF' & add 'Thus, after RCR: p. 4, l. 6.

Additional suggestions for further-reaching analyses: 

1. Candidates may chose different journals to publish if their assessment is based on RCR; a retrospective analysis if the journal profile of the faculty changes in the future would be fascinating.

Is the work clearly and accurately presented and does it cite the current literature?

Yes

If applicable, is the statistical analysis and its interpretation appropriate?

Yes

Are all the source data underlying the results available to ensure full reproducibility?

Yes

Is the study design appropriate and is the work technically sound?

Yes

Are the conclusions drawn adequately supported by the results?

Partly

Are sufficient details of methods and analysis provided to allow replication by others?

Yes

Reviewer Expertise:

Molecular Biology; Scientific Publishing in biomedical sciences. Co-author of DORA declaration

I confirm that I have read this submission and believe that I have an appropriate level of expertise to confirm that it is of an acceptable scientific standard.

# Peer review: Reviewer response for version 1

**DOI:** 10.5256/f1000research.29342.r72322

**Published:** 2020-10-14

**Authors:** Sandra L. Schmid

**Affiliations:** 1 Chan Zuckerberg Biohub, San Francisco, CA, USA; 2 Department of Cell Biology, University of Texas Southwestern Medical Center, Dallas, TX, USA

This is a very straight-forward comparison of the article-specific bibliometric RCR with the journal specific metric JIF. The results clearly support the conclusion that the JIFG is NOT a reliable metric of the impact of individual papers published in the journal and therefore should NOT be used as a tool to assess promotion and tenure. This is a very important, yet not unexpected, finding. It should be widely distributed and acted upon, just as the University of Bern has altered its policies.

Is the work clearly and accurately presented and does it cite the current literature?

Yes

If applicable, is the statistical analysis and its interpretation appropriate?

Yes

Are all the source data underlying the results available to ensure full reproducibility?

Yes

Is the study design appropriate and is the work technically sound?

Yes

Are the conclusions drawn adequately supported by the results?

Yes

Are sufficient details of methods and analysis provided to allow replication by others?

Yes

Reviewer Expertise:

I am a cell biologist and served as Chair of the Departments of Cell Biology at The Scripps Research Institute and UT Southwestern for a total of ~20 years, hiring and promoting faculty.  I was also a co-author and original signator of the DORA Proclamation.

I confirm that I have read this submission and believe that I have an appropriate level of expertise to confirm that it is of an acceptable scientific standard.
